# Human serum and platelet lysate are appropriate xeno-free alternatives for clinical-grade production of human MuStem cell batches

**DOI:** 10.1186/s13287-018-0852-y

**Published:** 2018-05-02

**Authors:** Charlotte Saury, Aurélie Lardenois, Cindy Schleder, Isabelle Leroux, Blandine Lieubeau, Laurent David, Marine Charrier, Laëtitia Guével, Sabrina Viau, Bruno Delorme, Karl Rouger

**Affiliations:** 1 Macopharma, Biotherapy Division, F-59420 Mouvaux, France; 2 PAnTher, INRA, École Nationale Vétérinaire, Agro-alimentaire et de l’alimentation Nantes-Atlantique (Oniris), Université Bretagne Loire (UBL), F-44307 Nantes, France; 3 IECM, INRA, Oniris, UBL, F-44307 Nantes, France; 4 Centre de Recherche en Transplantation et Immunologie UMR1064, INSERM, UBL, F-44093 Nantes, France; 5 Institut de Transplantation Urologie Néphrologie (ITUN), CHU Nantes, F-44093 Nantes, France; 6 Inserm UMS016, SFR François Bonamy, iPSC Core Facility, Nantes, France; 7 CNRS UMS 3556, Nantes, France; 8 Université de Nantes, Nantes, France; 9 CHU Nantes, Nantes, France; 10 Institut du thorax, INSERM, CNRS, Université de Nantes, Nantes, France; 11 Université de Nantes, F-44000 Nantes, France; 12 INRA, UMR 703, École Nationale Vétérinaire, Agroalimentaire et de l’Alimentation Nantes-Atlantique (Oniris), Route de Gachet, CS. 40706, F-44307 Nantes, France

**Keywords:** Adult stem cells, Platelet lysate, Human serum, Muscle disease, Cell therapy, Good manufacturing practice

## Abstract

**Background:**

Canine MuStem cells have demonstrated regenerative efficacy in a dog model of muscular dystrophy, and the recent characterization of human counterparts (hMuStem) has highlighted the therapeutic potential of this muscle-derived stem cell population. To date, these cells have only been generated in research-grade conditions. However, evaluation of the clinical efficacy of any such therapy will require the production of hMuStem cells in compliance with good manufacturing practices (GMPs). Because the current use of fetal bovine serum (FBS) to isolate and expand hMuStem cells raises several ethical, safety, and supply concerns, we assessed the use of two alternative xeno-free blood derivatives: human serum (HS) and a human platelet lysate (hPL).

**Methods:**

hMuStem cells were isolated and expanded in vitro in either HS-supplemented or hPL-supplemented media and the proliferation rate, clonogenicity, myogenic commitment potential, and oligopotency compared with that observed in FBS-supplemented medium. Flow cytometry and high-throughput 3′-digital gene expression RNA sequencing were used to characterize the phenotype and global gene expression pattern of hMuStem cells cultured with HS or hPL.

**Results:**

HS-supplemented and hPL-supplemented media both supported the isolation and long-term proliferation of hMuStem cells. Compared with FBS-based medium, both supplements enhanced clonogenicity and allowed for a reduction in growth factor supplementation. Neither supplement altered the cell lineage pattern of hMuStem cells. In vitro differentiation assays revealed a decrease in myogenic commitment and in the fusion ability of hMuStem cells when cultured with hPL. In return, this reduction of myogenic potential in hPL-supplemented cultures was rapidly reversed by substitution of hPL with HS or fibrinogen-depleted hPL. Moreover, culture of hMuStem cells in hPL hydrogel and fibrinogen-depleted hPL demonstrated that myogenic differentiation potential is maintained in heparin-free hPL derivatives.

**Conclusions:**

Our findings indicate that HS and hPL are efficient and viable alternatives to FBS for the preparation of hMuStem cell batches in compliance with GMPs.

**Electronic supplementary material:**

The online version of this article (10.1186/s13287-018-0852-y) contains supplementary material, which is available to authorized users.

## Background

In the last decade, the proof of concept of regenerative medicine for muscular dystrophies has been demonstrated using a variety of tissue-resident stem cell populations [1–8]. Preclinical findings have identified certain stem cell populations as promising candidates for the treatment of a variety of devastating and currently incurable diseases [9, 10]. Owing to their relative scarcity in adult tissues, generating sufficient numbers of cells for clinical applications involves a protracted ex vivo expansion period. Moreover, given that the in vitro environment can alter the intrinsic features of stem cells, special care is required to ensure the safety and reparative efficacy of cultured stem cells. It is therefore important to define the appropriate culture conditions required to produce cell batches for clinical use in compliance with good manufacturing practice (GMP) standards [11, 12].

Fetal bovine serum (FBS) has been traditionally used in vitro as a source of growth factors (GFs) and other elements essential for cell adhesion and proliferation [13]. However, its use has raised several concerns in recent years. FBS not only is a potential source of contamination by pathogens such as viruses, mycoplasmas, and prions, but can also increase cell immunogenicity as a result of the internalization of animal proteins during in vitro expansion [14–18]. In one cell therapy trial, antibodies against FBS proteins were detected in one out of six patients following cell infusion [19]. Other disadvantages include the significant batch-to-batch variation of FBS, and the possibility that the growing demand for FBS may soon exceed supply capacity [16, 20, 21]. Finally, the use of FBS raises several ethical concerns pertaining to serum harvesting practices and animal suffering [16, 22]. Taken together, these issues underscore the need for alternative clinically transferable in-vitro cell expansion protocols. Researchers have sought to develop fully defined media that can efficiently support cell proliferation, but with only partial success [23–25]. A variety of human blood derivatives including serum, platelet, and plasma concentrates have been tested, mainly using mesenchymal stem cells (MSCs). Some authors have proposed the use of human serum (HS) of both autologous and allogeneic origin [26–33], although studies have reported limited growth of MSCs cultured with allogeneic serum [18, 31]. While HS of autologous origin may be ideal to avoid potential immune cross-reactions, its use is limited by difficulties associated with the quality control of individual sera and the limited availability of large quantities required for clinical applications [25]. Other proposed alternatives include pooled human AB serum (i.e., from type AB donors) [26, 28, 33, 34] and human platelet lysate (hPL), which contains a higher concentration of GFs than other serum substitutes such as FBS and platelet-rich plasma (PRP) [35]. Recently, some studies even proposed to combine HS and hPL as culture medium supplements, based on demonstration of an improved proliferation rate of human bone marrow-derived MSCs compared to those obtained with HS alone [36, 37]. In the case of hPL, heterogeneous biological effects have been reported owing to significant inter-laboratory variability in the preparation processes (resulting in variability in platelet concentrate units and platelet concentrations), as well as the presence of leukocytes, anticoagulants, and activators [38–42]. Both HS and hPL have been widely reported to promote the proliferation of MSCs and to maintain their capacity for multilineage differentiation toward adipogenic, osteogenic, and chondrogenic lineages [26, 43–48], although certain hPL and PRP preparations may favor osteogenic differentiation [44, 49]. Few data have been generated using myogenic cell cultures. Culture of primary human myoblasts in hPL-supplemented media results in poor differentiation, perhaps due to the lack of unidentified myogenesis-promoting factors present in FBS [50]. Mainly due to the presence of platelet-derived growth factor (PDGF), PRP promotes the growth of human muscle-derived progenitor cells (hMDPCs), including preplated MDPCs, myoendothelial cells, and pericytes, while maintaining stemness [51]. Moreover, in vitro expansion in PRP-supplemented media causes no changes in the osteogenic, chondrogenic, or myogenic differentiation abilities of hMDPCs, or in their potential for in-vivo myofiber regeneration. The combination of PRP and decorin, an inhibitor of transforming growth factor beta-1 (TGF-β1), promotes proliferation and stimulates myogenic commitment in human myoblasts in vitro [52].

Based on their initial delayed adhesion properties, we isolated and characterized a population of adult stem cells (named MuStem cells) from healthy dog skeletal muscle, and demonstrated their efficacy following vascular delivery into the clinically relevant dog model of Duchenne muscular dystrophy (DMD), in which we observed persistent clinical stabilization and significant muscle repair [53–56]. More recently, we isolated and extensively characterized the human counterparts of these cells (hMuStem cells), and demonstrated their regenerative ability when delivered into injured muscle [57]. Taken together, these preclinical data point to hMuStem cells as a promising therapeutic candidate for patients with muscular dystrophy. However, the aforementioned findings were obtained using hMuStem cells generated in research-grade conditions, limiting their value as supportive data in submissions to regulatory agencies. In this study, we evaluated the effects on hMuStem cell features of two human blood derivatives used as FBS substitutes for the culture of stem cells. We evaluated the effects of pooled AB-HS (hereafter referred to as HS) and standardized, commercially available, clinical-grade hPL on hMuStem cell isolation and on the in-vitro proliferation rate, clonogenicity, differentiation ability, and phenotype of hMuStem cells.

## Methods

### Human skeletal muscle tissue

hMuStem cells were isolated from paravertebral muscle biopsies from 12 patients (aged 13–19 years) who were free of known muscular disease and underwent surgery for acute scoliosis at the Department of Pediatric Surgery of the Centre Hospitalier Universitaire (CHU), Nantes, France (Table 1). All patients provided written informed consent. All protocols were approved by the Clinical Research Department of the CHU (Nantes, France), in accordance with the rules of the French Regulatory Health Authorities. Biological sample banking was compliant with national guidelines for the use of human tissue for research (Permit numbers: MESR/DC-2010-1199; CPP/29/10).

**Table 1:** Donor age and sex, and medium type used for in vitro preparation of hMuStem cells

ID number	Donor age (years)	Sex	Nutrient used in medium		Sample name
			Isolation	Expansion	
♯1	15	Female	FBS	FBS	
♯2	15	Female			hMuStem cells^FBS^
♯3	18	Male			
♯4	13	Female	HS	HS	
♯5	15	Female			hMuStem cells^HS^
♯6	19	Male			
♯4	13	Female	hPL	hPL	
♯5	15	Female			hMuStem cells^hPL^
♯6	19	Male			
♯7	15	Male	HS	HS or hPL or FBS	hMuStem cells^HS/HS^ or hMuStem cells^HS/hPL^ or hMuStem cells^HS/FBS^
♯8	17	Male			
♯9	14	Female			
♯10	18	Male			
♯11	15	Female			
♯12	17	Female			

*FBS* fetal bovine serum, *HS* human serum, *hPL* human platelet lysate

### hMuStem cell isolation and culture

Muscle-derived cells (MDCs) were isolated using either the previously described research-grade protocol [57] or an adapted, GMP-compliant version thereof. Briefly, freshly obtained muscle biopsies were stored for up to 3 days in organ preservation solution (Macopharma, Mouvaux, France) supplemented with 2 IU/ml penicillin/0.1 mg/ml streptomycin/0.25 μg/ml amphotericin B (PSF; Sigma-Aldrich, St Louis, MO, USA). Muscle tissue was finely minced using forceps and scalpel, and was enzymatically digested (15 min, 37 °C) either with a mix of research-grade collagenase type VIII (2000 U/g of tissue; Sigma-Aldrich) and 0.2% hyaluronidase type-1S (Sigma-Aldrich), or with GMP-compliant collagenase (20 PZ/g of tissue; Coger, Paris, France). After centrifugation (100×*g*, 5 min), the supernatant was neutralized with F12 HAM medium (Invitrogen, Cergy-Pontoise, France) containing either 20% FBS (Eurobio, Les Ulis, France) for the research-grade protocol or 5% HS (EFS, Nantes, France) or 5% hPL (MultiPL’100; Macopharma) for the GMP-compliant protocol. The pellet was digested (30 min, 37 °C) with either 0.125% research-grade Pronase E (Sigma-Aldrich) or with GMP-compliant neutral protease (1.5 PZ/g of tissue; Coger). Digested tissue was then centrifuged (100×*g*, 5 min) and the supernatant was pooled with that obtained following the first enzymatic digestion step and centrifuged for an additional 15 min at 300×*g*. MDCs were obtained after sequential filtering through 100-μm, 70-μm, and 40-μm pore-diameter nylon meshes (BD Biosciences, Franklin Lakes, NJ, USA) and resuspended in F12 HAM medium containing either 2% FBS, 10% HS, or 10% hPL to assess cell number and viability using Trypan blue exclusion (VWR, Strasbourg, France). hMuStem cells were isolated after 6 days using a modified version of a previously described preplating technique [55, 57]. The cells were cultured under standard conditions (37 °C in a humidified atmosphere containing 5% CO_2_) in growth medium (GM) (Macopharma) supplemented with either 10% FBS/1% 10,000 UI/ml penicillin, 10 mg/ml streptomycin, and 25 μg/ml fungizone (amphotericin B) (PSF; Sigma-Aldrich, Saint Quentin-Fallavier, France)/10 ng/ml human recombinant basic fibroblast growth factor (bFGF; Miltenyi, Bergisch Gladbach, Germany)/25 ng/ml human recombinant epidermal growth factor (EGF; Miltenyi) (hMuStem cells^FBS^), 10% HS/1% PSF/10 ng/ml bFGF/2 ng/ml EGF (hMuStem cells^HS^), or 10% hPL/1% PSF/2 ng/ml EGF (hMuStem cells^hPL^). Heparin (5 IU/ml; Sanofi-Aventis, Frankfurt, Germany) was added to hPL-supplemented media to prevent gelation of the medium. Cells were seeded on CELLstart™ substrate (Invitrogen) at 2.5 × 10^3^ cells/cm^2^ and GM was replaced twice per week.

Three muscle biopsies (donors ♯4–♯6) were divided and hMuStem cells (hMuStem cells^HS^ and hMuStem cells^hPL^; Table 1) were isolated and cultured in parallel using medium supplemented with HS and hPL, respectively. Given the limited number of biopsies of sufficiently large size to divide into two parts, six other batches of hMuStem cells (donors ♯7–♯12) were isolated using HS and then cultured either with HS or hPL and analyzed at passage 3 (P3) or 4 (P4) (corresponding to 8.3 ± 0.3 and 12.0 ± 0.3 cumulative population doublings (CPDs), respectively). Expanded cells are referred to as hMuStem cells^HS/HS^ and hMuStem cells^HS/hPL^, respectively (Table 1).

### Growth factor concentrations

The contents of bFGF (#DFB50), EGF (#DEG00), insulin-like growth factor (IGF)-1 (#DG100), and hepatocyte growth factor (HGF, #DHG00) were measured in triplicate in two batches of hPL and two batches of HS by ELISA (Bio-techne, Minneapolis, MN, USA), following the manufacturer’s instructions. The absorbance was measured using an Infinite^®^ M200 PRO spectrometer (Tecan, Männedorf, Switzerland), and the results were analyzed using Magellan™ data analysis software (Tecan).

### In vitro proliferation kinetics

To define the proliferative properties of hMuStem cells cultured in different conditions, cells were seeded in triplicate at 2.5 × 10^3^ cells/cm^2^ and dissociated with TrypLE™ (Gibco, Life Technologies, Carlsbad, CA, USA) when the first of the three cell layers reached confluence. After centrifugation (410×*g*, 10 min), cells were resuspended in appropriate supplemented GM and the viable cell number was determined using Trypan blue staining. The number of population doublings (PDs) was calculated at each passage using the following formula: PD = log(*n*_f_/*n*_0_)/log(2), where *n*_0_ and *n*_f_ are the number of cells initially plated and the number of cells harvested at the end of the passage, respectively [58]. The CPD was calculated as the sum of the PD of the passage and those of the previous passages.

### Colony-forming unit assay

To evaluate the clonogenic potential of hMuStem cells, colony forming unit (CFU) assays were performed by plating hMuStem cells^HS^ and hMuStem cells^hPL^ at P4 (corresponding to 13.7–14.5 CPDs for highly proliferative batches and 9.6–11.8 CPDs for poorly proliferative batches, respectively) in triplicate in gelatin-coated dishes (Corning, New York, USA) at low density (< 5 cells/cm^2^) and in the appropriate supplemented GM. The medium was replaced every 4 days. After 8 days, cell layers were washed with phosphate buffered saline (PBS; PAA, Les Rumeaux, France), fixed with 4% paraformaldehyde (PFA; Sigma-Aldrich) for 10 min, and washed twice with PBS. Colonies were stained with May-Grünwald stain (Sigma-Aldrich) for 5 min at room temperature (RT), washed with distilled water, and counterstained with Giemsa (1:20 in PBS; Sigma-Aldrich). The frequency of CFUs was determined as the mean number of cell clusters containing at least 50 cells divided by the number of cells initially seeded, and expressed as a percentage.

### Flow cytometry analysis

Immunophenotype analyses were performed on expanded hMuStem cells^HS^ and hMuStem cells^hPL^ (P4). Cells were incubated (30 min at 4 °C in darkness) with antibody (Ab) against the appropriate cell-surface marker (Additional file 1: Table S1) and washed three times with PBS/2% HS. Fluorescence minus one control samples using isotype-matched Ab were used as negative controls for gating and analyses for multicolor labeling. Labeled cells were acquired using a FACS Aria flow cytometer (BD Biosciences). Data were analyzed using FlowJo software (FlowJo, Ashland, OR, USA). At least 10^4^ cells and 3 × 10^4^ cells were analyzed for single and multicolor labeling protocols, respectively.

### Immunofluorescence analysis

hMuStem cells cultured in the different supplemented GM were seeded at 3 × 10^4^ cells/cm^2^ on four-well μ-slides (Ibidi, Planegg, Germany) coated with CELLstart™ substrate. Twenty-four hours later, cell layers were fixed with 4% PFA (10 min, RT), permeabilized with 0.3% Triton X-100 (20 min, 4 °C), and incubated (60 min, RT) in blocking buffer (5% goat serum in PBS). Cells were then incubated with the appropriate Ab (listed in Additional file 2: Table S2). Finally, cells were counterstained (15 min, 37 °C) with 4′,6-diamidino-2-phenylindole (DAPI) fluorescent cell-permeable DNA probe (Life Technologies Ltd, Paisley, UK). Two large random fields were analyzed and over 331 cells were counted per sample using Fiji image analysis software [59].

### Reverse-transcription and real-time semiquantitative PCR

Total RNA was extracted using the RNeasy mini or micro kit following the manufacturer’s instructions (Qiagen, Santa Clara, CA, USA), quantified using a NanoDrop spectrophotometer (Labtech, Wilmington, DE, USA) after DNase treatment (Ambion, Austin, TX, USA), and converted to cDNA by reverse transcription as described previously [57]. Oligonucleotide primers used for semiquantitative RT-PCR analysis of gene expression were designed using Oligo Primer Analysis Software v.7 (Molecular Biology Insights Inc., Colorado Springs, CO, USA) and are listed in Additional file 3: Table S3. Data were normalized to mRNA levels of the housekeeping gene *RPS18* and were calculated using the 2^–∆Ct^ method.

### Digital gene expression sequencing

Total mRNA was extracted from hMuStem cells^HS^ (*n* = 3) and hMuStem cells^hPL^ (*n* = 3) at P4 and processed as described earlier. RNA integrity was determined using the Agilent Eukaryote Total RNA Nano kit with the 2100 Bioanalyzer (Agilent, Santa Clara, CA, USA). A high-throughput 3′ digital gene expression RNA-sequencing (DGE-seq) protocol was performed as described previously [60]. Briefly, the libraries were prepared from 10 ng of total RNA. The mRNA poly(A) tail was tagged with universal adapters, well-specific barcodes, and unique molecular identifiers (UMIs) during template-switching reverse transcription. Barcoded cDNAs from multiple samples were then pooled, amplified, and tagmented using a transposon-fragmentation approach that enriches for 3′ ends of cDNA. A library of 350–800 base pairs (bp) was run on an Illumina HiSeq 2500 using a TruSeq Rapid SBS kit (Illumina, San Diego, CA, USA). Sequencing-derived data were deposited in the Gene Expression Omnibus [61] and can be accessed using GEO accession number GSE99085. Read pairs used for analysis fulfilled the following criteria: all 16 bases of the first read must have quality scores of at least 10 and the first six bases must correspond exactly to a designed well-specific barcode. The second reads were aligned to RefSeq human mRNA sequences (*hg19*, obtained from the UCSC Genome Browser) using bwa version 0.7.4 4 with the nondefault parameter “-l 24”. Reads mapping to several positions in the genome were filtered out from the analysis. Digital gene expression (DGE) profiles were generated by counting the number of unique UMIs associated with each RefSeq gene for each sample.

### Differential gene expression

Differential expression analysis was performed using the DESeq2 Bioconductor package version 1.14.1 [62]. The data were analyzed using a multifactor design to take into account the differences between patients while estimating the effect attributable to culture conditions. Genes showing significant differences in expression between hMuStem cells^HS^ and hMuStem cells^hPL^ were identified using a fold-change threshold ≥ 2 and adjusted *p* < 0.05.

### In vitro myogenic differentiation

For myogenic differentiation, hMuStem cells were seeded at 3 × 10^4^ cells/cm^2^ on 24-well plates and cultured in media supplemented with either 10% HS, 10% hPL, or 10% FBS for 2 weeks, after which HS, hPL, or FBS was replaced with 1% FBS (differentiation medium (DM)). After 4 days, cultures were fixed in 4% PFA, and incubated with 5% Triton X-100 (30 min, 4 °C), 20% goat serum in PBS (20 min, RT), and finally anti-human sarcomeric myosin heavy chain isoform (sMyHC) Ab (1:500; Developmental Studies Hybridoma Bank/DSHB, Iowa City, IA, USA) for 60 min at 37 °C. Specific Ab binding was then visualized using AlexaFluor^®^ 488-coupled secondary Ab (1:500; Invitrogen) and nuclei were counterstained with DRAQ5 (1:1000; Biostatus, Loughborough, UK). The fusion index (FI) was determined as the ratio of nuclei within sMyHC^+^ myotubes (≥ 2 nuclei) to the total number of nuclei. Two random fields in each of three replicate wells were analyzed and at least 651 nuclei per well were considered.

The behavior of hMuStem cells was also assessed in coculture experiments with dystrophic cells (D7 cell line; kindly provided by D. Yaffe from primary culture of an adult 129REJ dy/dy mouse). After expansion in different culture conditions, hMuStem cells and D7 cells were mixed at a ratio of 5:1 for a final density of 3 × 10^4^ cells/cm^2^ in Dulbecco’s Modified Eagle Medium (DMEM; Invitrogen)/10% FBS/1% PSF for 1 day, after which FBS was replaced with 2% horse serum. After 4 days, multinucleated cells were visualized as described earlier by immunolabeling for sMyHC. Hybrid myotubes were detected using specific human lamin A/C Ab (1:500; Abcam, Cambridge, UK) and combined with AlexaFluor^®^ 555-coupled secondary Ab (1:200; Invitrogen).

### Western blot assay

For protein extraction, cells were homogenized in RIPA lysis buffer containing 150 mM NaCl, 50 mM Tris–HCl, pH 7.4, 1% Nonidet-P40, 1% glycerol, 1 mM EDTA, and protease inhibitors using the Precellys (2 × 10 s, 6500 rpm; Ozyme, France). Homogenates were centrifuged at 14,000×*g* to pellet debris (15 min, 4 °C). The protein concentration was determined using a BCA protein assay (Sigma-Aldrich). Fifteen micrograms of proteins from cell homogenate were resolved by sodium dodecyl sulfate polyacrylamide gel electrophoresis (SDS-PAGE) on 4–12% polyacrylamide gels (NuPage, Life Technologies, Illkirch, France) and electroblotted onto nitrocellulose membranes (Protran BA 83; GE Healthcare Life Sciences, Velizy-Villacoublay, France) using a Bio-Rad^®^ liquid blotting system at 30 mA for 2 h. The membranes were blocked using 50% blocking buffer (Odyssey^®^; Li-Cor Biosciences, Lincoln, NE, USA) in PBS (60 min, RT) and incubated overnight at 4 °C with primary Abs against sMyHC (1:1000, DSHB) and GAPDH (1:1000, CliniSciences, Nanterre, France). After washing with Tween 0.1% in PBS, the blots were incubated with fluorophore-conjugated anti-mouse and anti-rabbit secondary antibody. Equal protein loading was verified through GAPDH labeling and Ponceau red staining of the membranes. Western blot bands were scanned with Odyssey^®^.

### In vitro adipogenic and osteogenic differentiation

hMuStem cells (P4) were seeded in triplicate at 3 × 10^4^ cells/cm^2^ and cultured in appropriate supplemented GM for 1 day, after which they were incubated in specific adipogenic and osteogenic cell induction media for 14 and 21 days, respectively, as described previously [63]. Adipogenic differentiation was determined by the detection of small neutral lipid vesicles after staining with Nile Red and quantified using AdipoRed™ Assay Reagent (Lonza, Walkersville, MD, USA) following the manufacturer’s instructions. Osteogenic differentiation was determined by calcium deposit staining with Alizarin Red S (ARS; Sigma) and quantified by optical density (OD) measurement after ARS dissolution, measured for each replicate well after extraction using 20% methanol/10% acetic acid (250 μl/cm^2^, 15 min at 450 nm), as described previously [64]. Total RNA from undifferentiated and differentiated hMuStem cells was harvested for RT-qPCR analysis. Expression levels of the peroxisome proliferator activated receptor gamma (*PPARγ*) and the integrin binding sialoprotein (*IBSP*) genes were determined to assess adipogenic and osteogenic differentiation, respectively. Primers are listed in Additional file 3: Table S3.

### Statistical analysis

Results are presented as the mean ± standard deviation (SD) where technical replicates are presented, and as the mean ± SEM when the number of samples is > 3. Statistical analyses were performed using R software (3.3.2 version). Data were tested for normality and independence before analysis, as described previously [65]. Statistical differences were calculated using a linear mixed effect (LME) model considering the donor as the random term, followed by Tukey’s multiple comparison post-hoc test [66]. The Wilcoxon signed-rank test or Friedman test followed by Dunn’s multiple comparison test were used where appropriate for nonindependent or non-normal data. Differences were considered significant at *p* < 0.05.

## Results

### The in vitro growth ability of hMuStem cells is unchanged by substitution of FBS with either HS or hPL

**Fig. 1:**
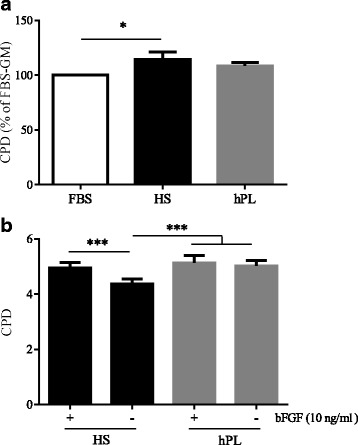
Proliferative behavior of hMuStem cells^FBS^ cultured in vitro in HS-supplemented or hPL-supplemented medium. **a** hMuStem cells^FBS^ (*n* = 3, donors #1–#3) cultured for 14 days in growth medium (GM) supplemented with either 10% FBS, 10% HS, or 10% hPL. Proliferation rates determined as mean cumulative population doubling (CPD) number, expressed as percentage of corresponding values obtained for FBS-GM (*p < 0.05, Friedman test). **b** hMuStem cells^HS^ (*n* = 3, donors #4–#6) cultured for 6 days in GM supplemented with either 10% HS or 10% hPL, in presence or absence of 10 ng/ml bFGF. Proliferation rates determined as CPD (***p < 0.001, LME model followed by Tukey’s post-hoc test). FBS fetal bovine serum, HS human serum, hPL human platelet lysate, bFGF basic fibroblast growth factor

To assess the validity of HS and hPL as alternatives to FBS in hMuStem cell culture, we first quantified proliferation rates of three hMuStem cells^FBS^ batches cultured in GM supplemented with either 10% HS or 10% hPL, and compared these results with those obtained in GM supplemented with 10% FBS. After 12 days, the number of CPDs recorded was 11.3 ± 0.7 and 10.7 ± 0.9 for hMuStem cells^FBS^ cultured with HS and hPL, respectively, compared with 9.9 ± 0.9 for those cultured in FBS (Fig. 1a). Compared with hMuStem cells^FBS^ cultured with FBS, those cultured with HS exhibited a significantly higher proliferation rate (*p* < 0.05), while that of cells cultured with hPL was unchanged. No statistical differences were found between hMuStem cells^FBS^ cultured with HS and hPL, indicating that both human supplements support hMuStem cell expansion. Interestingly, the absence of 10 ng/ml bFGF in HS-supplemented medium negatively affected the proliferation of hMuStem cells^FBS^, as evidenced by a 33.7% decrease in CPD, an effect not observed in hMuStem cells^FBS^ cultured in hPL-supplemented medium (Fig. 1b). Using ELISA, we determined that HS and hPL exhibit, in a reproducible way, similar concentrations of IGF-1 and HGF (of the order of 120–132 ng/ml and 1104–1325 pg/ml, respectively) but distinct ones for bFGF and EGF, these being 11.6 and 1.8 times superior in hPL respectively (Additional file 4: Table S4). Based on these findings, two clinical-grade GMs were used in all subsequent in-vitro experiments, supplemented either with 10% HS/10 ng/ml bFGF/2 ng/ml EGF (referred to as HS) or 10% hPL/2 ng/ml EGF/5 IU/ml heparin (referred to as hPL).

### Both HS-supplemented and hPL-supplemented media support the isolation and growth of hMuStem cells

**Fig. 2:**
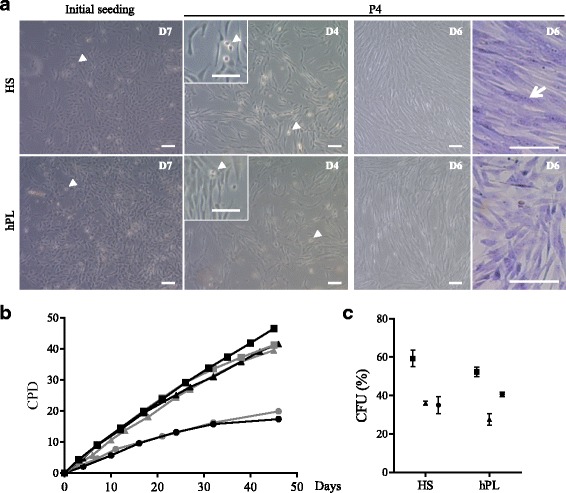
In vitro morphological and behavioral features of hMuStem cells^HS^ and hMuStem cells^hPL^. **a** Phase-contrast microscopy images of hMuStem cells^HS^ (upper panels) and hMuStem cells^hPL^ (lower panels) 7 days after initial seeding (left panels) and after four passages (P4). In exponential phase of amplification, both culture types consisted primarily of spindle-shaped cells, as well as small number of poorly adherent round cells (arrowheads). At day 6 from P4 (i.e., when cells reached confluence), May-Grünwald Giemsa staining of multinucleated cells revealed spontaneous fusion in hMuStem cells^HS^ (arrow, top right panels). In contrast, no multinucleated cells observed in hMuStem cells^hPL^ (bottom right panels). Scale bar, 100 μm. **b** Long-term proliferation kinetics of cultured hMuStem cells^HS^ (black) and hMuStem cells^hPL^ (gray) from three independent donors. **c** Frequency of colony-forming units (CFU) in hMuStem cells^HS^ and hMuStem cells^hPL^ at P4. Data presented as mean ± SD for each donor. Donors #4, #5, and #6 represented by squares, triangles, and circles, respectively. HS human serum, hPL human platelet lysate, D day, P passage, CPD cumulative population doubling

Muscle biopsies collected from three young donors were each divided to evaluate the isolation and expansion of MuStem cells in HS-GM and hPL-GM (hereafter referred to as hMuStem cells^HS^ and hMuStem cells^hPL^, respectively) (Table 1). Both cell types began to proliferate after 5–7 days, resulting in the formation of pseudoclonal cultures consisting predominantly of thin fusiform cells, as well as poorly adherent round cells (Fig. 2a, left panels, arrowhead). Over successive passages, the nonconfluent cells displayed a characteristic thin spindle-like morphology, and refringent round cells were consistently observed (Fig. 2a, middle panels, arrowhead in insert), as described previously for hMuStem cells^FBS^ [57]. Compared with hMuStem cells^HS^, which tended to align rapidly and occasionally fused spontaneously when highly confluent (Fig. 2, top right panels, arrow), hMuStem cells^hPL^ were smaller and more refractive, and tended to generate multilayer cultures without fusing, regardless of confluence and alignment (Fig. 2, bottom right panels). Next, we examined the direct effect of nutritive supplements on long-term hMuStem cell proliferation. Cells were maintained in their respective GM for up to 40 days and CPD was calculated at each passage. hMuStem cells^HS^ and hMuStem cells^hPL^ derived from donors ♯4 and ♯5 homogeneously generated between 31.0–33.8 and 31.4–33.5 CPDs by day 32, respectively (Fig. 2b). By contrast, at the same time point only 15.7 and 16.2 CPDs were recorded for donor ♯6-derived hMuStem cells^HS^ and hMuStem cells^hPL^, respectively, indicating more limited proliferative potential. Despite the heterogeneity in intrinsic proliferative potential between cell batches, which is likely associated with patient history, no differences in CPD were observed between cells cultured using the two human nutritive substitutes. The clonogenic potential of the six hMuStem cell batches was evaluated at P4. The frequency of CFUs corresponded to 43.4 ± 7.9% and 40.2 ± 7.1% for hMuStem cells^HS^ and hMuStem cells^hPL^, respectively, suggesting comparable potential to form colonies in both conditions (Fig. 2c).

### hMuStem cells^HS^ and hMuStem cells^hPL^ share a similar mesenchymal/perivascular phenotype but have distinct myogenic signatures

**Fig. 3:**
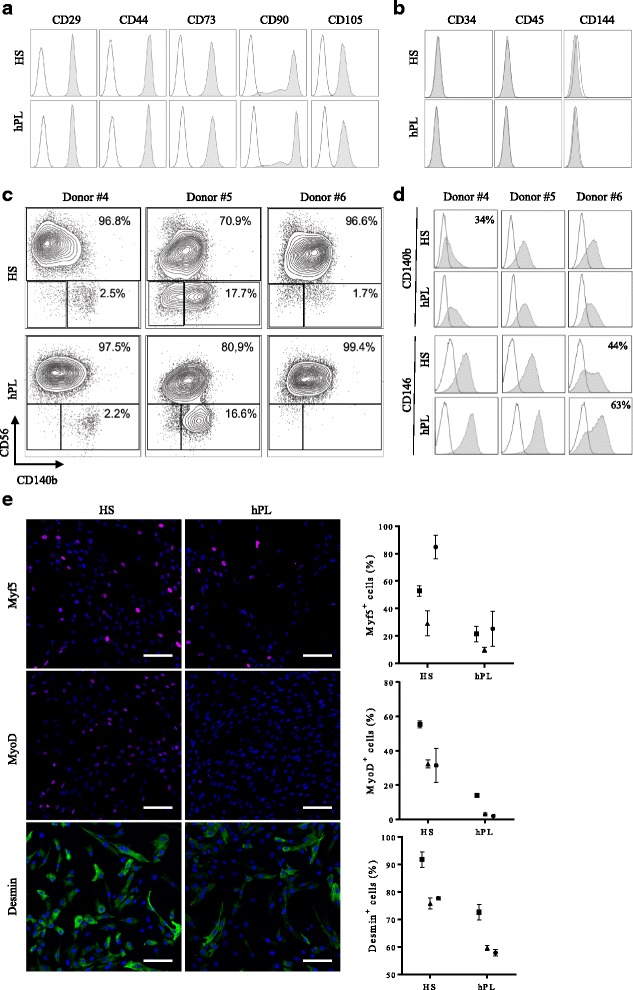
Phenotypic profile of hMuStem cells^HS^ and hMuStem cells^hPL^. Flow cytometry comparison of (**a**) mesenchymal (CD29, CD44, CD73, CD90 and CD105) and (**b**) hematopoietic (CD34, CD45) and endothelial (CD144) cell lineage markers in hMuStem cells^HS^ and hMuStem cells^hPL^. Results of one representative donor out of three independent donors presented. Myogenic (CD56) and perivascular (CD140b and CD146) cell lineage markers analyzed either in (**c**) entire viable cell population or (**d**) CD56^+^ cell subsets. Results presented for three independent donors: when significant but below 100, percentage of positive cells indicated in upper right corner of corresponding histograms. **e** Immunocytochemistry analysis of myogenic regulatory factors (MRFs) Myf5, MyoD, and desmin in hMuStem cells^HS^ and hMuStem cells^hPL^ at P4. Representative pictures presented. Nuclei counterstained with DAPI (blue). Proportions of positive cells recorded for three independent donors presented. Donors #4, #5, and #6 represented by squares, triangles, and circles, respectively. Data presented as mean ± SD for each donor. Scale bar, 100 μm. HS human serum, hPL human platelet lysate

hMuStem cells^FBS^ have been described as early myogenic progenitor cells of perivascular origin [57]. We investigated whether hMuStem cells cultured in HS-GM or hPL-GM shared a similar phenotype. Flow cytometry analysis revealed that hMuStem cells^HS^ and hMuStem cells^hPL^ (*n* = 3, P4) abundantly expressed (> 99%) the typical MSC markers CD29, CD44, CD73, CD90, and CD105 (Fig. 3a), but were negative for classical hematopoietic (CD34, CD45) [67] and endothelial (CD144) cell markers (Fig. 3b), in agreement with previous observations in hMuStem cells^FBS^ [57]. As expected, the fraction of neural cell adhesion molecule (NCAM)/CD56^+^ cells was significant in all batches, with CD56^+^ cells accounting for 71–99% of the entire population (Fig. 3c). The percentage of CD56^−^ cells was always slightly reduced in hMuStem cells^hPL^ versus hMuStem cells^HS^; the CD56^−^ population was even undetectable in one sample (donor ♯6). In both hMuStem cells^HS^ and hMuStem cells^hPL^, two populations were identified among CD56^−^ cells, depending on their expression of CD140b. A significant proportion of CD56^−^/CD140b^+^ cells expressed CD146, regardless of culture conditions (data not shown). Culture in HS-GM and hPL-GM poorly affected the expression by CD56^+^ cells of the well-known perivascular cell markers CD140b and CD146 (Fig. 3d). All CD56^+^ cells expressed moderate CD140b expression, except in one sample (donor ♯4, HS condition) where only 34% of the cells were positive. In two samples (donors ♯4 and ♯5), CD56^+^ cells exhibited homogeneous expression of CD146 in both culture conditions, while in one sample (donor ♯6) the percentage of CD146^+^ cells was 44% in hMuStem cells^HS^ and 63% in hMuStem cells^hPL^. Taken together, these results indicate that hMuStem cells cultured in HS-GM or hPL-GM share a similar phenotype, although culture in hPL resulted in a slightly less heterogeneous cell population. The main source of variation was the donor. The myogenic profile of hMuStem cells^HS^ and hMuStem cells^hPL^ was further analyzed based on their expression of the myogenic regulatory factors (MRFs) Myf5 and MyoD, and of muscle-specific intermediate filament desmin. We found that the proportions of Myf5^+^, MyoD^+^, and desmin^+^ cells were consistently smaller in hMuStem cells^hPL^ versus hMuStem cells^HS^, which exhibited decreases of 2.6–3.0-fold, 4.0–15.4-fold, and 1.3–1.4-fold, respectively (Fig. 3e). These data suggest that commitment to the myogenic lineage is reduced in hMuStem cells isolated and expanded in hPL-GM versus HS-GM.

**Fig. 4:**
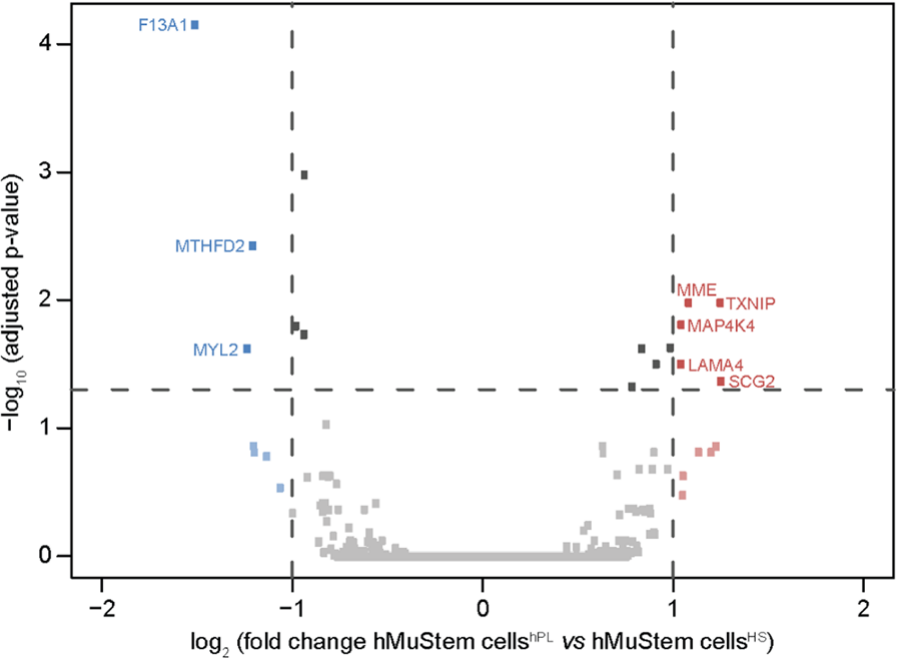
Differential gene expression in hMuStem cells^HS^ and hMuStem cells^hPL^ detected by DGE sequencing. The 14,936 expressed genes represented by squares. Vertical dashed lines indicate cutoff values for significant fold-change (≤ 0.5 or ≥ 2). Blue squares represent downregulated genes (log_2_ (fold-change) ≤ −1), red squares represent upregulated genes (log_2_ (fold-change) ≥ 1). Horizontal dashed line represents adjusted *p*-value cutoff (adjusted *p* < 0.05). Gene names specified for eight genes showing significant differences in expression between hMuStem cells^HS^ and hMuStem cells^hPL^

To further evaluate the effects of the nutritive substitutes HS and hPL on global gene expression in hMuStem cells, a DGE-seq protocol was performed. Of the 14,936 genes detected in at least one out of the six cell batches (hMuStem cells^HS^, *n* = 3; hMuStem cells^hPL^, *n* = 3), few differentially expressed genes were identified (Additional file 5: Table S5). The volcano plot in Fig. 4 illustrates the general gene expression pattern in the two hMuStem cell preparations. Statistically significant differences in expression were assessed using a 2-fold change threshold associated with adjusted *p* < 0.05. Only eight genes fulfilled these conditions, indicating very limited differences in the effects of the two human nutritive substitutes on gene expression in hMuStem cells. It should be noted that these genes did not correspond to any of the classical targets used to define cell lineage, including the genes included in the panel used for cytometry analysis in the present study. Five genes, corresponding to membrane metallo-endopeptidase (*MME*), thioredoxin interacting protein (*TXNIP*), mitogen-activated protein kinase 4 (*MAP4K4*), laminin subunit alpha 4 (*LAMA4*), and secretogranin II (*SCG2*), were upregulated in hMuStem cells^hPL^ versus hMuStem cells^HS^. Previous studies have described heparin-induced induction of *TXNIP* and an interaction between heparin and *LAMA4* [68, 69]. As such, the upregulation of these genes may be a result of the presence of heparin in hPL-GM. On the other hand, genes encoding the coagulation factor XIII A chain (*F13A1*), methylenetetrahydrofolate dehydrogenase 2 (or NADP-dependent methylenetetrahydrofolate dehydrogenase 2-like protein) (*MTHFD2*), and the myogenic marker myosin light chain 2 (*MYL2*) were downregulated in hMuStem cells^hPL^ versus hMuStem cells^HS^. Together, the results of cytometry, immunocytochemistry, and DGE-seq analysis show that while the mesenchymal/perivascular phenotype is maintained in hMuStem cells expanded in HS-GM or hPL-GM, hMuStem cells^hPL^ tend to display reduced myogenic commitment compared with hMuStem cells^HS^.

### hMuStem cells^HS^ and hMuStem cells^hPL^ are oligopotent cells with distinct myogenic commitment capabilities

**Fig. 5:**
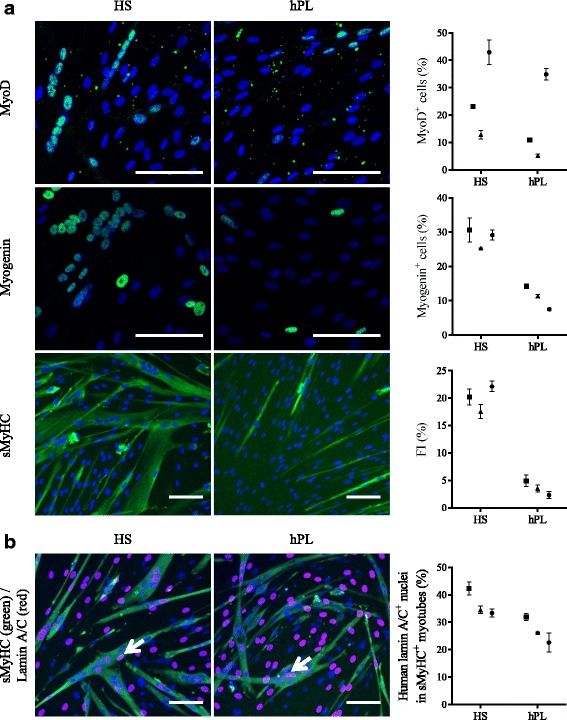
Myogenic differentiation potential of hMuStem cells^HS^ and hMuStem cells^hPL^. **a** Myogenic differentiation potential of hMuStem cells isolated and expanded either with HS (hMuStem cells^HS^) or hPL (hMuStem cells^hPL^) determined at P4 (*n* = 3, donors #4–#6). Cells expressing myogenic markers MyoD and myogenin counted and fusion index (FI) calculated as percentage of nuclei identified in sarcomeric myosin heavy chain-positive (sMyHC^+^) myotubes. **b** Contribution of hMuStem cells to myogenesis evaluated in coculture experiments with mouse D7 cells. Number of human lamin A/C^+^ nuclei (arrow) counted within hybrid sMyHC^+^ myotubes. Nuclei counterstained with DAPI or DRAQ5 (blue). Donors #4, #5, and #6 represented by squares, triangles, and circles, respectively. Data presented as mean ± SD for each donor. Scale bar, 100 μm. HS human serum, hPL human platelet lysate

Like canine MuStem cells [55], in vitro characterization of hMustem cells^FBS^ has revealed a high myogenic differentiation potential [57]. After 4 days in DM, immunocytochemistry revealed that the proportions of MyoD^+^ and myogenin^+^ cells were consistently smaller in hMuStem cells^hPL^ versus hMuStem cells^HS^, in which decreases of 1.2–2.5-fold and 2.1–3.9-fold, respectively, were observed (Fig. 5a). Thus, as observed in cells expanded in GM, the proportion of cells positive for myogenic markers in DM was lower in hMuStem cells isolated and expanded in hPL than in HS. The fusion index (FI), corresponding to the percentage of nuclei inside multinucleated cells expressing sarcomeric myosin heavy chain (sMyHC), reached 17.6–22.2% in hMuStem cells^HS^ and 2.3–4.9% in hMuStem cells^hPL^, suggesting that the ability to fuse is reduced in cells cultured in hPL versus HS (4.1–9.7-fold change), an effect that may be related to the reduced myogenic commitment of the former.

To further investigate these apparent differences in fusion rates, coculture experiments were performed with the D7 dystrophic mouse cell line. Hybrid myotubes (i.e., containing human and mouse nuclei) (Fig. 5b, arrow) were observed in cultures of both hMuStem cells^HS^ and hMuStem cells^hPL^, confirming the ability of these cells to fuse. Human nuclei located in sMyHC^+^ myotubes accounted for 33.4–42.3% and 22.6–32.0% of all nuclei in D7 cocultures with hMuStem cells^HS^ and hMuStem cells^hPL^, respectively. Moreover, cells cultured with hPL displayed reduced fusion ability compared with those cultured with HS (1.3–1.5-fold change), even though they fused more efficiently when cocultured with the D7 cell line than when cultured alone in DM (FI: 22.6–32.0% versus 2.3–4.9%). Taken together, these results suggest that hMuStem cells^hPL^ are less capable of spontaneously differentiating into myogenic cells than hMuStem cells^HS^, which showed a differentiation potential similar to that of hMuStem cells^FBS^ [57]. Nonetheless, hMuStem cells^hPL^ were capable of efficiently fusing and generating myotubes when cocultured with the D7 cell line.

**Fig. 6:**
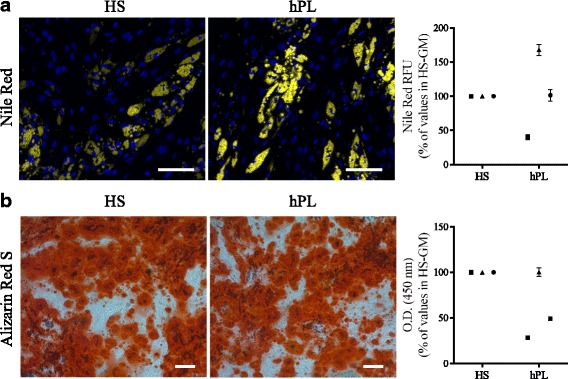
Adipogenic and osteogenic differentiation potential of hMuStem cells^HS^ and hMuStem cells^hPL^. (**a**) Adipogenic and (**b**) osteogenic differentiation potential of hMuStem cells isolated and expanded with either HS or hPL (*n* = 3, donors #4–#6) evaluated at P4. **a** Intracytoplasmic lipid vesicles, visualized by Nile Red staining, quantified after 14 days in adipogenic induction medium. Nuclei counterstained with DAPI (blue). Staining quantified using AdipoRed™ assay. **b** After 21 days in specific induction medium, osteogenesis evaluated by Alizarin Red S staining (ARS) of calcium deposits. Staining quantified by optical density (O.D.) measurement after ARS dissolution. Scale bar, 100 μm. Donors #4, #5, and #6 represented by squares, triangles, and circles, respectively. Data presented as mean ± SD for each donor. HS human serum, hPL human platelet lysate, GM growth medium, RFU relative fluorescence units

hMuStem cells^FBS^ have been described previously as oligopotent cells based on their ability to differentiate into osteogenic and adipogenic lineages as well as the myogenic lineage [57]. Given the aforementioned differences observed in the myogenic commitment capabilities of hMuStem cells^HS^ and hMuStem cells^hPL^, we next investigated their ability to differentiate into osteogenic and adipogenic lineages. After 14 days in adipogenic induction medium, fluorescent Nile Red staining revealed the presence of cytoplasmic accumulations of small lipid vesicles in cells from all batches, regardless of isolation and culture conditions (Fig. 6a). Relative quantification of Nile Red staining revealed no significant differences between culture conditions. Alizarin Red S staining revealed the formation of calcium deposits in all cultures after 21 days in osteogenic induction medium (Fig. 6b). Relative quantification revealed that the numbers of calcium deposits in hMuStem cells^hPL^ were comparable to or less than those observed in hMuStem cells^HS^ cultures (Fig. 6b). These results suggest that both hMuStem cells^HS^ and hMuStem cells^hPL^ maintain their ability to differentiate into adipogenic and osteogenic lineages, even though hPL may have a partial suppressive effect on osteogenic differentiation, as also observed for myogenic differentiation.

### Effect of hPL on the oligopotency of hMuStem cells^HS^

The results already described, based on MuStem cells obtained from only three donors, suggest that culture of hMuStem cells with hPL reduces the myogenic commitment capability of these cells, as well as their ability to differentiate into myogenic and osteogenic lineages. We hypothesized that the differences observed were due to the type of nutritive substitute used during in-vitro expansion rather than intrinsic properties of MuStem cells^HS^ and MuStem cells^hPL^. To confirm this hypothesis, six additional batches of hMuStem cells^HS^ (P4) were cultured for two passages in either HS-GM or hPL-GM. The experiments described earlier for hMuStem cells^HS^ and hMuStem cells^hPL^ were repeated for each of these batches (hereafter referred to as hMuStem cells^HS/HS^ and hMuStem cells^HS/hPL^, respectively) (Table 1).

**Fig. 7:**
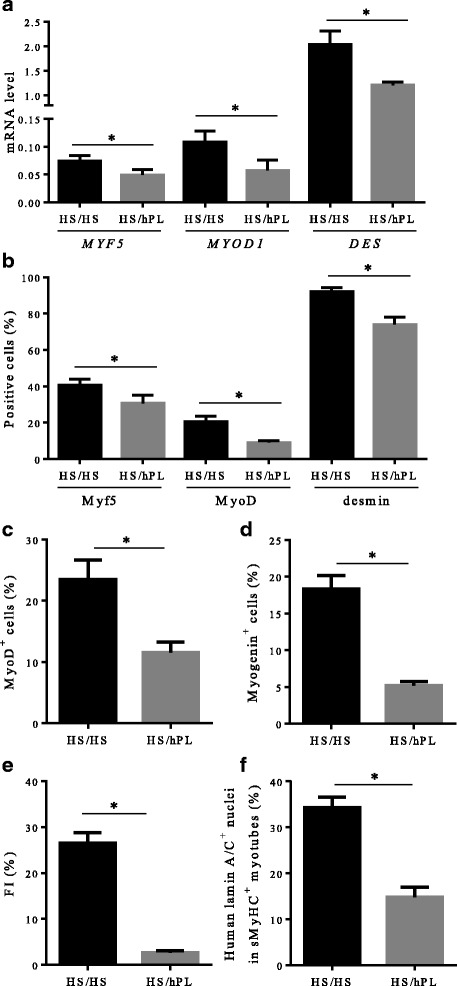
Influence of hPL on myogenic differentiation potential of hMuStem cells^HS^. hMuStem cells^HS^ (*n* = 6, donors #7–#12) at P3–4 cultured for two passages with HS (hMuStem cells^HS/HS^) or hPL (hMuStem cells^HS/hPL^) and resulting myogenic pattern evaluated. Myogenic commitment assessed by determining (**a**) mRNA expression levels of MRFs *MYF5*, *MYOD1*, and *DES*, as well as (**b**) proportion of cells positive for each of these markers. Myogenic differentiation potential assessed by quantification of cells expressing (**c**) MyoD and (**d**) myogenin, and by determining (**e**) fusion index (FI), defined as percentage of nuclei identified in sarcomeric myosin heavy chain-positive (sMyHC^+^) myotubes. (**f**) Influence of hPL on contribution of hMuStem cells to myogenesis evaluated in coculture experiments with mouse D7 cells by quantification of human lamin A/C^+^ nuclei within hybrid sMyHC^+^ myotubes. Data presented as mean ± SEM (*p < 0.05, Wilcoxon matched-pairs signed rank test). HS human serum, hPL human platelet lysate

**Fig. 8:**
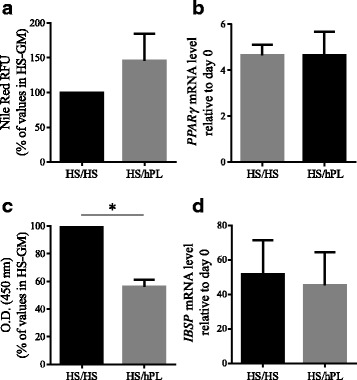
Influence of hPL on adipogenic and osteogenic differentiation potential of hMuStem cells^HS^. hMuStem cells^HS^ (*n* = 6, donors #7–#12) at P3–4 cultured for two passages with HS (hMuStem cells^HS/HS^) or hPL (hMuStem cells^HS/hPL^) and their potential to differentiate into adipogenic and osteogenic lineages in specific induction media evaluated. **a, b** Presence of intracytoplasmic lipid vesicles, visualized by Nile Red staining, assessed after 14 days in adipogenic induction medium. (**a**) Staining quantified using the AdipoRed™ assay and (**b**) mRNA levels of *PPARγ* quantified. **c, d** After 21 days in specific induction medium, osteogenesis evaluated by Alizarin Red S staining (ARS) of calcium deposits. (**c**) Staining quantified by optical density (O.D.) measurement after ARS dissolution and (**d**) mRNA levels of *IBSP* quantified. Data presented as mean ± SEM (*p < 0.05, Wilcoxon matched-pairs signed rank test). HS human serum, hPL human platelet lysate, RFU relative fluorescence units, GM growth medium, *PPARγ* peroxisome proliferator activated receptor gamma, *IBSP* integrin binding sialoprotein

As shown in Fig. 7a, mRNA expression levels of *MYF5*, *MYOD1*, and *DES* were 1.5-fold, 1.9-fold, and 1.7-fold lower, respectively, in hMuStem cells^HS/hPL^ versus hMuStem cells^HS/HS^ (*p* < 0.05). At the protein level, 40.6 ± 3.2%, 20.7 ± 2.9%, and 92.1 ± 2.3% of hMuStem cells^HS/HS^ were positive for Myf5, MyoD, and desmin, respectively, compared with only 30.7 ± 4.5%, 9.1 ± 1.2%, and 74.0 ± 4.0% of hMuStem cells^HS/hPL^ (Fig. 7b; *p* < 0.05). After 4 days in DM, MyoD and myogenin were expressed in 23.5 ± 3.2% and 18.3 ± 1.8% of hMuStem cells^HS/HS^, respectively, whereas only 11.6 ± 1.7% and 5.2 ± 0.5% of hMuStem cells^HS/hPL^ expressed these proteins (Fig. 7c, d; *p* < 0.05). In line with our observations in hMuStem cells^HS^ and hMuStem cells^hPL^, the FI values recorded for hMuStem cells^HS/HS^ and hMuStem cells^HS/hPL^ were 26.5 ± 5.6% and 2.6 ± 1.1%, respectively (Fig. 7e; *p* < 0.05). In comparison, hMuStem cells^HS/FBS^ (i.e., cells cultured in FBS as a control group in parallel; see Table 1) exhibited FI values of 15.1 ± 6.3%, showing that replacement of the FBS used routinely until then with HS does not compromise the differentiation capability but even allows it to increase slightly, unlike replacement with hPL which limits it strongly (*p* < 0.05). In D7 coculture experiments performed with hMuStem cells^HS/HS^ and hMuStem cells^HS/hPL^, we detected 34.3 ± 2.3% and 14.8 ± 2.2%, respectively, of human nuclei in sMyHC^+^ myotubes (i.e., a lower percentage in cells cultured in hPL versus HS) (Fig. 7f; *p* < 0.05). Analysis of adipogenic differentiation potential revealed no significant differences in lipid vesicle staining or *PPARγ* mRNA expression between culture conditions, confirming the maintenance of adipogenic potential in both hMuStem cells^HS/HS^ and hMuStem cells^HS/hPL^ (Fig. 8a, b). Alizarin Red S staining revealed reduced mineralization in hMuStem cells^HS/hPL^ versus hMuStem cells^HS/HS^ (Fig. 8c), confirming the impact of hPL on the osteogenic potential of hMuStem cells, whether included before hMuStem cell isolation or added afterward. However, we observed no significant differences between hMuStem cells^HS/hPL^ and hMuStem cells^HS/HS^ in mRNA expression of the *IBSP* gene, a classical marker of osteogenic differentiation (Fig. 8d), suggesting a moderate effect of hPL. Overall, these results corroborate the reduced myogenic commitment capability previously observed in hMuStem cells isolated and cultured with hPL, but indicate no major effect of culture condition on adipogenic and osteogenic differentiation potential.

### Heparin and fibrinogen exert inhibitory effects on myogenic differentiation

**Fig. 9:**
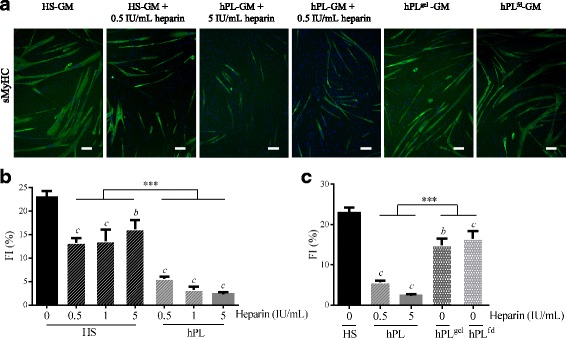
Influence of heparin and heparin-free hPL-supplemented media on myogenic potential of hMuStem cells. hMuStem cells^HS^ cultured for 6 days in HS-GM or hPL-GM in absence or presence of increasing doses of heparin (0.5–5 IU/ml), or in heparin-free hPL alternatives, and then seeded for evaluation using myogenic differentiation assay. **a** Immunolabeling of sMyHC performed after 4 days in myogenic differentiation medium DM. Nuclei counterstained with DRAQ5 (blue). Scale bar, 100 μm. Fusion index (FI) determined in (**b**) three independent cell batches cultured with HS or hPL in presence of increasing doses of heparin, or in (**c**) heparin-free hPL alternatives; heparin-free hPL that was allowed to form a nutritive gel over the cell layer (hPL^gel^), or heparin-free fibrinogen-depleted hPL (hPL^fd^). Data presented as mean ± SEM (^b^*p* < 0.01, ^c^*p* < 0.001 versus HS-GM; ***p < 0.001; LME model followed by Tukey’s post-hoc test). HS human serum, hPL human platelet lysate, GM growth medium, sMyHC sarcomeric myosin heavy chain

In terms of content, one major difference between the two human nutritive substitutes used in the present study is the presence in hPL of plasma fibrinogen and of heparin, which is required to avoid gelation of the medium. In light of previous studies suggesting that the heparin concentration may affect stem cell proliferation and/or differentiation [70], we investigated the effects of heparin on myogenic commitment in MuStem cells. hMuStem cells^HS^ were cultured in the presence of increasing doses of heparin (0.5–5 IU/ml), with the highest dose corresponding to that found in hPL-GM. Culture for 6 days in GM in the presence of heparin resulted in higher population doublings (PDs) that ranged from 5.13 ± 0.34 to 5.25 ± 0.21 compared with 4.86 ± 0.23 in GM without heparin, showing a positive, dose-independent effect of heparin on the proliferation rate of hMuStem cells^HS^ (Additional file 6: Figure S1). Concomitantly, these cultures gave rise to fewer and smaller myotubes, even at the lowest dose, as illustrated in Fig. 9a for the 0.5 IU/ml dose. The FI of hMuStem cells^HS^ was 22.9 ± 1.4%, and ranged from 13.2 ± 1.0 to 16.2 ± 1.9% in the presence of heparin, revealing a negative, dose-independent effect of heparin on the fusion ability of hMuStem cells^HS^ (Fig. 9b). Interestingly, in a parallel experiment in which hMuStem cells^HS^ were cultured for 6 days in hPL containing the same range of doses of heparin, the drastically reduced FI value observed in cells cultured with the same dose of heparin as found in hPL-GM (5 IU/ml, 2.3 ± 0.4%) did not differ significantly from that observed for the lowest dose (0.5 IU/ml, 5.6 ± 0.4%) (Fig. 9a, b). We also found that addition of heparin to HS-GM did not repress myogenic differentiation to the same extent as when added to hPL. Thus, FI values for heparin-containing HS-GM were significantly higher than those recorded for hPL-GM, independent of heparin dose.

We next investigated the myogenic differentiation potential of hMuStem cells in heparin-free hPL-GM. hMuStem cells^HS^ were plated on CELLstart™ substrate-coated plates and covered with nutritive gel (hPL^gel^ condition). In this condition, we recorded an FI of 14.9 ± 1.6%, as compared with 5.6 ± 0.4% in the presence of 0.5 IU/ml heparin, indicating a significant inhibitory effect of heparin on myogenic differentiation (*p* < 0.001) (Fig. 9a, c). Nonetheless, these FI values were significantly lower than those obtained with HS-GM (22.9 ± 1.4%, *p* < 0.001), suggesting that the reduction in myogenic differentiation may not be entirely attributable to heparin. We next investigated the effects of culture in fibrinogen-depleted hPL-GM (hPL^fd^), produced by the addition of a calcification step to the hPL production process, circumventing the need for heparin supplementation. Myogenic differentiation potential was evaluated after one passage of 6 days in either HS-GM, hPL-GM, or hPL^fd^-GM (Fig. 9a, c). Compared with hMuStem cells^HS^ (FI = 22.9 ± 1.4%), the fusion capacity of hMuStem cells^HS/hPL^ remained low (FI = 2.3 ± 0.4%), while that of hMuStem cells^HS/hPLfd^ was significantly higher (FI = 16.5 ± 1.9%, *p* < 0.001) but still significantly lower than that of hMuStem cells cultured in HS-GM (*p* < 0.01). These results indicate that myogenic differentiation potential is maintained in heparin-free hPL, but is lower than that observed in cells cultured in HS. These findings confirm the role of heparin in the suppressive effect of hPL on myogenic differentiation of hMuStem cells, but do not rule out the potential contribution of other factors.

### The suppressive effect of hPL on the myogenic differentiation potential of hMuStem cells is reversible

**Fig. 10:**
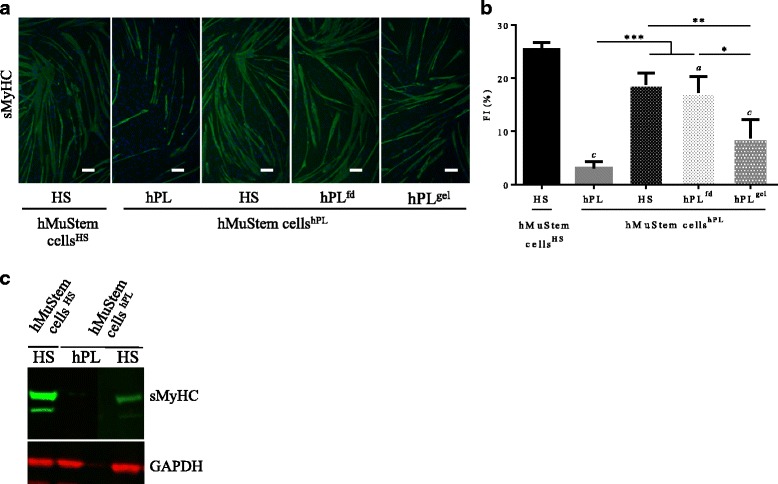
Reversal of suppressive effect of hPL on myogenic potential of hMuStem cells. hMuStem cells^hPL^ cultured up to P3 in appropriate GM and then passaged to HS-GM or heparin-free hPL-GM (hPL^fd^ or hPL^gel^), then myogenic differentiation assessed. **a** sMyHC immunolabeling performed after 4 days of culture in fusion-promoting low-serum medium. Nuclei counterstained with DRAQ5 (blue). Scale bar, 100 μm. **b** Fusion index (FI) in six independent cell batches. **c** Western blot analyses of sMyHC and GAPDH protein abundance in hMuStem cells^HS^ maintained in HS-GM, as well as hMuStem cells^hPL^ cultured with either hPL-GM or HS-GM. Data presented as mean ± SEM (^a^*p* < 0.05, ^c^*p* < 0.001 versus HS; *p < 0.05, **p < 0.01, ***p < 0.001; LME model followed by Tukey’s post-hoc test). HS human serum, hPL human platelet lysate, hPL^gel^ heparin-free hPL that was allowed to form a nutritive gel over the cell layer, hPL^fd^ heparin-free fibrinogen-depleted hPL, sMyHC sarcomeric myosin heavy chain, GAPDH glyceraldehyde 3-phosphate dehydrogenase

To complete our characterization of the suppressive effect of hPL on the myogenic potential of hMuStem cells, hMuStem cells^hPL^ (P3) were cultured for the fourth passage (5 or 6 days) with either hPL, HS, or heparin-free hPL (hPL^fd^ or hPL^gel^). Myogenic differentiation was assessed at the end of P4 (Fig. 10). As described earlier, hMuStem cells^hPL^ displayed a significantly lower fusion capacity than hMuStem cells^HS^ (FI, 3.1 ± 1.3% versus 25.4 ± 1.3%). Interestingly, FI values recorded for hMuStem cells^hPL^ cultured in HS-GM and hPL^fd^-GM (18.7 ± 2.3% and 17.2 ± 3.1%, respectively) were higher than those of cells cultured with hPL (*p* < 0.001) (Fig. 10a, b). This indicates that the suppressive effect of hPL on the myogenic potential of hMuStem cells^hPL^ is reversible. These data obtained from sMyHC immunolabeling were confirmed by western blot analysis, showing a lack of detection of the protein from hMuStem cells^hPL^ cultured in hPL-GM in opposition to the strong abundance detected from hMuStem cells^HS^ as well as the presence of a moderate expression from hMuStem cells^hPL^ cultured in HS-GM (Fig. 10c). Interestingly, no significant differences in FI values were observed between hMuStem cells^HS^ and hMuStem cells^hPL/HS^. However, the FI for hMuStem cells^hPL^ cultured in hPL^gel^ did not differ significantly from that observed in the initial hPL-GM. Together, these results suggest that the impairment of myogenic commitment in hMuStem cells^hPL^ can be reversed by passage in HS-GM or in hPL^fd^-GM.

## Discussion

Over the past 6 years, several studies have demonstrated the clinical efficacy of MuStem cell treatment to repair muscle tissue, highlighting the potential of this muscle-derived stem cell population in cell-based therapies [53–56]. hMuStem cells have been previously isolated, expanded, and characterized using FBS-supplemented growth culture media [57]. However, the identification of suitable alternatives for the production of hMuStem cell batches has become a key research focus, given the inherent risks associated with the use of a xenogeneic serum source and likely future shortages with increasing demand [21].

Potential FBS substitutes evaluated to date include HS and platelet derivatives such as PRP and hPL, which efficiently support and generally promote in-vitro proliferation of MSCs derived from bone marrow [26, 31, 47, 71], adipose tissue [28], and other tissue sources [34]. The few studies that have evaluated the viability of HS and platelet derivatives in myogenic cell cultures have reported conflicting results [50–52, 72]. Here, we demonstrate that supplementation of human MuStem cell primary cultures with either 10% HS or 10% hPL results in a similar proliferation rate and greater clonogenic potential of MuStem cells when compared to those obtained with 10% FBS supplementation [57], corroborating the pro-proliferative effects of these two nutritive supplements. Owing to the GF content of HS and hPL, the use of these supplements allowed for a reduction in the concentration of EGF in GM, and for the removal of bFGF in the case of hPL-supplemented media, with no negative impact on proliferation. Given the interest in limiting the number of products required for cell batch preparation, HS and hPL show significant promise for the production of hMuStem cell products.

While the expression of classical myogenic (CD56), mesenchymal (CD29, CD44, CD73, CD90, CD105), and pericyte (CD140b, CD146) cell markers was maintained in the hMuStem cells^HS^ and hMuStem cells^hPL^, the latter contained a lower proportion of cells expressing the myogenic regulatory factors Myf5 and MyoD, and intermediate filament desmin. These observations suggest reduced myogenic commitment capabilities in the presence of hPL, and are supported by the lower FI recorded for hMuStem cells^hPL^, indicating a reduced ability to differentiate in vitro (when cultured in a myogenesis-promoting medium) into the myogenic cell lineage. By contrast, the rate of differentiation of hMuStem cells^HS^ was comparable to that described previously for hMuStem cells^FBS^ [57]. Nonetheless, hMuStem cells^hPL^ were able to fuse efficiently (albeit to a lesser extent than hMuStem cells^HS^) when cocultured with the D7 dystrophic cell line, evoking that hMuStem cells^hPL^ may participate in in-vivo muscle regeneration via regeneration signals generated by other cells in a pathological context. Mitogen-activated protein 4 kinase 4 (Map4k4) has been recently shown to negatively regulate skeletal muscle differentiation of C2C12 cells in a Myf5-dependent manner [73]. Using DGE RNA-sequencing technology and RT-qPCR analysis, we found that *MAP4K4* and *MYF5* genes were upregulated and downregulated, respectively, in hMuStem cells^hPL^ versus hMuStem cells^HS^, suggesting that the Map4k4-related signaling pathway may contribute to the suppressive effect of hPL on myogenic differentiation of hMuStem cells.

A key difference between HS and hPL is the presence of plasma coagulation factors such as fibrinogen and other platelet-derived factors [26, 42] in hPL, which necessitates the addition of anticoagulants to prevent coagulation and clot formation when used to supplement calcium-containing culture media. Heparin, which is widely used in transfusion medicine and is available as an authorized drug suitable for injection, is also currently used in the expansion of hPL-supplemented hMSCs in clinical trials [74]. Clinical-grade production of purified fractionated heparin, an animal-derived biological product, is highly regulated (monographs in the European Pharmacopeia and good manufacturing practice (GMP)). Nonetheless, the concentration and quality of heparin used in cell culture is critical to maintain the optimal proliferative and ex-vivo differentiation abilities of MSCs. These reports support the finding of the present study, in which the addition of heparin reduced the myogenic commitment and differentiation capabilities of cultured hMuStem cells^HS^, even at the lowest concentration of heparin used to prevent gelation of the medium. The use of fibrin gel as a 3D-nutritive matrix for MSC expansion has been proposed to preclude the need for heparin supplementation [75]. However, in our experimental conditions, expansion of hMuStem cells^HS^ in fibrin gel resulted in a drastic reduction in proliferation rate. Another alternative method we tested was to culture hMuStem cells^HS^ beneath a nutritive fibrin matrix. This produced cells with the spindle-shape morphology typical of hMuStem cells, albeit with a slightly reduced proliferation rate and an intermediate myogenic potential, confirming a suppressive effect of heparin supplementation on myogenic potential. Another strategy to circumvent the need for heparin supplementation is to generate serum-converted hPL by depleting fibrinogen, either by adding CaCl_2_ or activating thrombin [42, 76], or by mechanical depletion [77]. When the fibrinogen content was reduced by the addition of CaCl_2_ (hPL^fd^), hMuStem cells^HS/hPLfd^ displayed an intermediate myogenic potential, highly suggesting that heparin in hPL contributes to the observed reduction in the myogenic potential of hMuStem cells. Interestingly, passage of hMuStem cells^hPL^ in HS-supplemented medium resulted in a rate of differentiation comparable to that observed in hMuStem cells^HS^, indicating that the reduction in myogenic commitment capabilities caused by heparin-containing hPL is reversible. Passage of hMuStem cells^hPL^ in hPL^fd^-GM produced similar results, but only partially restored myogenic potential. The hPL^gel^ condition was the least effective in restoring the myogenic potential of hMuStem cells^hPL^. Taken together, our results describe an effective two-step alternative for hMuStem cell^hPL^ expansion that produces cells with a myogenic potential comparable to that of hMuStem cells^HS^. One possible application of this method is to carry out the isolation procedure and the initial stages of in-vitro expansion using hPL-GM, thereby maintaining MuStem cells in their proliferative state, and subsequently switching to heparin-free hPL-GM (hPL^fd^) for the final stages of production to increase the myogenic potential. The absence of spontaneous fusion of hMuStem cells during in-vitro expansion could be advantageous in the context of clinical applications, providing greater intra-batch homogeneity in terms of cell phenotype, commitment capabilities, and morphology. Morphology is of particular interest, given that the presence of enlarged differentiated cells may impair biodistribution and tissue dissemination and provoke clotting during in-vivo injection [9]. Further studies will be required to assess the in-vivo regenerative potential of hMuStem cells after expansion in different culture conditions. Also, the presence of other factors in hPL or HS media that may have an additional inhibiting or promoting effect on myogenesis could be considered, as recently suggested for bFGF and TGF-β1 [50–52, 78–80].

Key considerations when choosing a culture supplement include its effect on cell features, its compatibility with the large-scale production processes required for clinical applications, and availability. The production of hPL requires more steps than that of HS, including successive freezing/thawing steps, the elimination of debris by centrifugation and/or filtration, and the addition of reagents such as CaCl_2_, thrombin, or heparin. This increases the likelihood of variability between laboratories and means that a greater level of control is required to ensure adherence to GMP [28]. Standardization of the amplification of cell therapy products is also crucial to achieve the necessary level of reproducibility in the manufacturing process. In our study, we used standardized, characterized, clinical-grade hPL obtained from 70 donors and manufactured in 10-L batches. The established reproducibility of the production process ensured markedly less batch-to-batch variability than that associated with FBS production. Moreover, the reproducibility of cytokine content and cell proliferation efficacy have been tested in human MSCs cultured in hPL and documented by the manufacturer. The HS used in this study was produced in batches of 3 L using a standardized process including viral inactivation, obtained from 15–20 donors selected according to standard blood donation requirements. Each sample is biologically classified as a blood donation and new serologies are realized at least within 60 days for every donor to secure donation.

## Conclusion

The present findings indicate that HS and hPL are suitable for the isolation and expansion of hMuStem cells for use in clinical applications. Our findings represent an important step forward in the development of a clinically compliant process for the production of hMuStem cells, and their subsequent testing in future clinical trials.

## Additional files

Additional file 1: **Table S1.** List of antibodies used for hMuStem cell characterization by flow cytometry analysis (PDF 16 kb)

Additional file 2: **Table S2.** List of antibodies used for hMuStem cell characterization by immunocytochemistry analysis (PDF 11 kb)

Additional file 3: **Table S3.** List of primers used for RT-qPCR analysis (PDF 14 kb)

Additional file 4: **Table S4.** Growth factor concentrations (PDF 42 kb)

Additional file 5: **Table S5.** The UMI counts for each gene in each sample normalized by dividing by the sum of UMI counts across all genes in the same sample. Normalized UMI counts expressed in UPM (UMI counts for one gene per 1,000,000 UMI counts for all genes) (XLSX 8238 kb)

Additional file 6: **Figure S1.** Influence of heparin on hMuStem cell proliferation rates. hMuStem cells^HS^ cultured for 6 days in HS-GM without heparin or with increasing doses of heparin (0.5–5 IU/ml). Population doublings (PDs) determined in three independent cell batches (^a^*p* < 0.05, ^b^*p* < 0.01 versus HS-GM without heparin; LME model followed by Tukey’s post-hoc test) (PDF 34 kb)

## Abbreviations

ARS: Alizarin Red S
bFGF: Basic fibroblast growth factor
cDNA: Complementary DNA
CFU: Colony forming unit
CHU: Centre Hospitalier Universitaire
CPD: Cumulative population doubling
DAPI: 4′,6-Diamidino-2-phénylindole
DGE: Digital gene expression
DM: Differentiation medium
EGF: Epidermal growth factor
ELISA: Enzyme-linked immunosorbent assay
FBS: Fetal bovine serum
FI: Fusion index
GF: Growth factor
GM: Growth medium
hMDPC: Human muscle-derived progenitor cell
hPL: Human platelet lysate
hPL^df^: Fibrinogen-depleted hPL
HS: Human serum
MDC: Muscle-derived cell
MGG: May-Grünwald Giemsa
MRF: Myogenic regulatory factor
MSC: Mesenchymal stromal cell
MyHC: Myosin heavy chain
PBS: Phosphate buffered saline
PCR: Polymerase chain reaction
PD: Population doubling
PDGF: Platelet-derived growth factor
PFA: Paraformaldehyde
PPARγ: Peroxisome proliferator activated receptor gamma
PRP: Platelet-rich plasma
PSF: Penicillin/streptomycin/amphotericin B
RPS18: Ribosomal protein S18
RT: Room temperature
UMI: Unique molecular identifier
