# Supplementary material for: Human serum and platelet lysate are appropriate xeno-free alternatives for clinical-grade production of human MuStem cell batches
Source: Stem Cell Res Ther. 2018 May 2;9:128. doi: 10.1186/s13287-018-0852-y (PMC5932844; doi:10.1186/s13287-018-0852-y)
Supplement: Supplementary file 1 — Table S1. List of antibodies used for hMuStem cell characterization by flow cytometry analysis (PDF 16 kb) [file 13287_2018_852_MOESM1_ESM.pdf]

Table S1. List of antibodies used for hMuStem cell characterization by flow cytometry analysis

Primary antibody	Compagny and reference	Corresponding isotype
CD13-PE	BD Biosciences, 555392	Mouse IgG1-PE
CD29-PE	BD Biosciences, 555443	Mouse IgG1-PE
CD34-PE	BD Biosciences, 345802	Mouse IgG1-PE
CD44-PE	BD Biosciences, 555479	Mouse IgG2b-PE
CD45-PE	BD Biosciences, 555483	Mouse IgG1-PE
CD56-AF647	BD Biosciences, 562413	Mouse IgG1-AF647
CD73-PE	BD Biosciences, 550257	Mouse IgG1-PE
CD90-PE	BD Biosciences, 555596	Mouse IgG1-PE
CD105-PE	BD Biosciences, 560839	Mouse IgG1-PE
CD140b-PE	BD Biosciences, 558821	Mouse IgG2a-PE
CD144-PE	BD Biosciences, 560410	Mouse IgG1-PE
CD146-PE-Cy7	BD Biosciences, 562135	Mouse IgG1-PE-Cy7
Mouse IgG1 control-PE	BD Biosciences, 555749	N/A
Mouse IgG2b control-PE	BD Biosciences, 555743	N/A
Mouse IgG1 control-PE	BD Biosciences, 557714	N/A
Mouse IgG1 control-PE-Cy7	BD Biosciences, 557872	N/A
Mouse IgG2a control-PE	R&D Systems, IC003P	N/A
