# Supplementary material for: Human serum and platelet lysate are appropriate xeno-free alternatives for clinical-grade production of human MuStem cell batches
Source: Stem Cell Res Ther. 2018 May 2;9:128. doi: 10.1186/s13287-018-0852-y (PMC5932844; doi:10.1186/s13287-018-0852-y)
Supplement: Supplementary file 2 — Table S2. List of antibodies used for hMuStem cell characterization by immunocytochemistry analysis (PDF 11 kb) [file 13287_2018_852_MOESM2_ESM.pdf]

Table S2. List of antibodies used for hMuStem cell characterization by immunocytochemistry analysis

Primary antibody	Compagny and reference	Dilution	Incubation time and temperature	Secondary antibody
MYF5	Abcam, Ab125078	1:200	60 min, 37°C	GAR Alexa 488
MYOD	Dako, M3512	1:10	60 min, 37°C	GAM Alexa 488
MYOGENIN	DSHB, F5D	1:10	60 min, 37°C	GAM Alexa 488
DESMIN	Dako, M0706	1:50	60 min, RT	GAM Alexa 488
sMyHC	DSHB, MF20	1:500	60 min, 37°C	GAM Alexa 488
LAMIN A/C	Abcam, Ab108595	1:250	60 min, 37°C	GAR Alexa 550
