# Supplementary material for: Human serum and platelet lysate are appropriate xeno-free alternatives for clinical-grade production of human MuStem cell batches
Source: Stem Cell Res Ther. 2018 May 2;9:128. doi: 10.1186/s13287-018-0852-y (PMC5932844; doi:10.1186/s13287-018-0852-y)
Supplement: Supplementary file 3 — Table S3. List of primers used for RT-qPCR analysis (PDF 14 kb) [file 13287_2018_852_MOESM3_ESM.pdf]

Table S3. List of primers used for RT-qPCR analysis

Gene symbol	Type		Sequence	Product (bp)	Accession number
RPS18	Housekeeping gene	For	5'-ACCAAGAGGGCGGGAGAA-3'	85	NM_022551.2
		Rev	5'-CTGGGATCTTGTACTGGCGTG-3'		
MYF5	Myogenic genes	For	5'-CCACGACCAACCCCAACCA-3'	122	NM_005593.2
		Rev	5'-TCCCGGCAGGCTATAGTAGT-3'		
MYOD1		For	5'-TTGCCACAACGGACGACT-3'	120	NM_002478.4
		Rev	5'-AGTGCTCTTCGGGTTTCAGG-3'		
DES		For	5'-TCCAGTCCTACACCTGCGAGA-3'	214	NM_001927.3
		Rev	5'-TCCAGGGCCATCTTCACGTT-3'		
PPARG	Adipogenic gene	For	5’-ACTTCTCCAGCATTTCTACTCC-3’	217	NM_138712.3
		Rev	5’-ACACGACATTCAATTGCCAT-3’		
IBSP	Osteogenic gene	For	Proprietary information	88	qHSACED0002933
		Rev	Proprietary information		
