# Supplementary material for: Human serum and platelet lysate are appropriate xeno-free alternatives for clinical-grade production of human MuStem cell batches
Source: Stem Cell Res Ther. 2018 May 2;9:128. doi: 10.1186/s13287-018-0852-y (PMC5932844; doi:10.1186/s13287-018-0852-y)
Supplement: Supplementary file 4 — Table S4. Growth factor concentrations (PDF 42 kb) [file 13287_2018_852_MOESM4_ESM.pdf]

Table S4. Growth factor concentrations

	bFGF (pg/mL)		IGF-1 (ng/mL)		EGF (pg/mL)		HGF (pg/mL)	
	mean	SD	mean	SD	mean	SD	mean	SD
hPL batch #1	163.2	10.1	108.3	2.1	2051.6	173.7	1113.5	11.9
hPL batch #2	163.0	5.9	131.6	6.3	1990.8	60.1	1094.6	27.8
HS batch #1	13.8	1.8	139.3	4.0	1016.9	18.6	1264.7	22.3
HS batch #2	14.5	0.7	124.2	6.3	1167.1	62.5	1384.6	46.5
