# Supplementary figures and images for: Human serum and platelet lysate are appropriate xeno-free alternatives for clinical-grade production of human MuStem cell batches
Source: Stem Cell Res Ther. 2018 May 2;9:128. doi: 10.1186/s13287-018-0852-y (PMC5932844; doi:10.1186/s13287-018-0852-y)

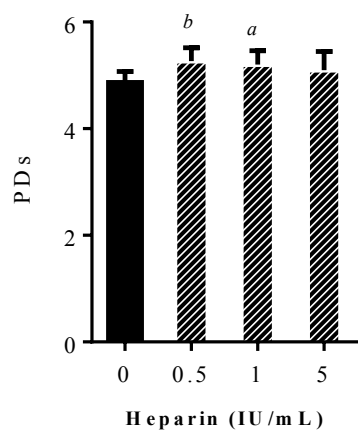

Supplement: Supplementary file 6 — Figure S1. Influence of heparin on hMuStem cell proliferation rates. hMuStem cellsHS cultured for 6 days in HS-GM without heparin or with increasing doses of heparin (0.5–5 IU/ml). Population doublings (PDs) determined in three independent cell batches (ap < 0.05, bp < 0.01 versus HS-GM without heparin; LME model followed by Tukey’s post-hoc test) (PDF 34 kb) [file 13287_2018_852_MOESM6_ESM.pdf]
